# Single-cell screening of multiple biophysical properties in leukemia diagnosis from peripheral blood by pure light scattering

**DOI:** 10.1038/s41598-017-12990-4

**Published:** 2017-10-04

**Authors:** David Dannhauser, Domenico Rossi, Mimmo Ripaldi, Paolo A. Netti, Filippo Causa

**Affiliations:** 1 Center for Advanced Biomaterials for Healthcare@CRIB, Istituto Italiano di Tecnologia (IIT), Largo Barsanti e Matteucci 53, 80125 Naples, Italy; 2 BMT Unit, Department of Pediatric Hemato-Oncology, Santobono-Pausilipon Hospital, Via Posillipo, 226, 80123 Naples, Italy; 3 Interdisciplinary Research Centre on Biomaterials (CRIB) and Dipartimento di Ingegneria Chimica, dei Materiali e della Produzione Industriale, Università degli Studi di Napoli “Federico II”, Piazzale Tecchio 80, 80125 Naples, Italy

## Abstract

Histology and histopathology are based on the morphometric observations of quiescent cells. Their diagnostic potential could largely benefit from a simultaneous screening of intrinsic biophysical properties at single-cell level. For such a purpose, we analyzed light scattering signatures of individual mononuclear blood cells in microfluidic flow. In particular, we extracted a set of biophysical properties including morphometric (dimension, shape and nucleus-to-cytosol ratio) and optical (optical density) ones to clearly discriminate different cell types and stages. By considering distinctive ranges of biophysical properties along with the obtained relative cell frequencies, we can identify unique cell classes corresponding to specific clinical conditions (p < 0.01). Based on such a straightforward approach, we are able to discriminate T-, B-lymphocytes, monocytes and beyond that first results on different stages of lymphoid and myeloid leukemia cells are presented. This work shows that the simultaneous screening of only three biophysical properties enables a clear distinction between pathological and physiological mononuclear blood stream cells. We believe our approach could represent a useful tool for a label-free analysis of biophysical single-cell signatures.

## Introduction

The relevance of cell investigation has a long history. The first microscopic observations in the seventeenth century already pointed out that the structure and morphology of a cell hold an enormous potential in permitting a fast identification of different cell types and stages^1^. In fact, most measurable cell properties can be related to their health conditions^2^. For instance, abnormal dimensions of the nucleus and/or the nucleus-to-cytosol ratio (n/c-ratio) are evident indicators of malignant transformations in white blood cells^3–6^, while the presence of cytosolic anomalies or aberrations generally indicates a cell suffering^7^. Such nucleus irregularities can be affected by DNA reorganizations during malignant cell transformations causing a changed biophysical optical property^8–10^.

Biophysical cells properties can be very useful in clearly distinguishing physiological from pathological cells and therefore support hematologists in choosing adequate therapies^11,12^. In particular, the screening of mechanical and morphometric cell properties has recently shown to give important information to distinguish cell classes and stages^13–16^. In fact, the imaging of cell stretching can identify cell stages such as pluripotency^13^, whereas the n/c-ratio of cells can help to identify circulating tumor cells^14^. Furthermore, classes of white blood cells, can be recognized according to their dimensions and responses of third harmonic generation microscopy^15^. Another recent work matches bright- and dark-field images to recognize cell stages according to their DNA amount^16^.

However, when dealing with a large number of cells, it is difficult to focus on the distinctive biophysical properties of a single cell of interest^17^. Such a drawback implies a loss of diagnostic power, when a few or even one single cell in a bulk of cells is sought. Therefore, an upcoming interest to microfluidic based approaches is emerging, allowing highly precise single-cell screening opportunities^18–20^. As example, the ability to catch circulating tumor cells in the blood stream, is an issue of great interest for the diagnosis of hidden tumorigenic events^21^. Furthermore, the screening of leukemic minimal residual disease is of extreme importance for patient follow-up and pharmacologic treatments^22,23^. Therefore, the capability of a precise single-cell analysis is of great demand, whereas a high throughput lab-on-a-chip method permitting a highly accurate single-cell screening is still missing^24^.

Currently, the modern cellular diagnostic paradigm is based on flow cytometry, where blood stream cells are determined by expressions of surface molecules, called Cluster of Differentiation (CD)^25–27^. Antibodies (Ab), coupled with fluorescent molecules, selectively bind CD and thereby classify cells^28^, making the analysis of biophysical properties less relevant for cell classifications^29^. Although such an approach is used as a standard detection system to identify many types of cells and to perform differential diagnosis, the need of fluorescent Ab-labeling is complex, time consuming, destructive and expensive^30,31^. Moreover, specialized personal is required to prepare, perform and interpret the measurement. The flow cytometry -which allows a very high cell throughput- yields only a rough measurement about the investigated cell complexities and dimensions, whereas no direct information of size and density of the investigated cell nucleus can be gained. To allow more sophisticated biophysical property investigations of single cells in microfluidic flows, slower interrogation times are needed, requiring new approaches to align cells. For instance, the use of viscoelastic polymers can help to simplify the alignment process, maintaining full preservation of cell morphology and vitality^27^. In fact, to circumvent flow cytometry limitations, much effort has been recently devoted to label-free approaches, with particular attention to single-cell analysis^32^.

In this context, we report a simple and non-destructive light scattering profile (LSP) analysis of individual mononuclear blood stream cells in-flow, which is able to investigate multiple biophysical properties of cells, in order to distinguish physiological from pathological cell stages. A distinctive optical signature for each viscoelastically aligned cell is obtained, without using any type of cell labelling. Such approach allows the simultaneous readout of multiple biophysical properties deriving from such a signature and, therefore, an immediate characterization of unknown cells. In fact, out of these signatures we can retrieve biophysical properties such as dimension, density and n/c-ratio of a single cell. We investigated peripheral blood mononuclear cell (PBMC) samples of several healthy donors to prove the efficiency of our completely label-free screening. We found distinct differences in biophysical properties of cells for each class of PBMC and clearly distinguished T- from B-lymphocytes as well as from monocytes. We report detailed investigations of 8 different cases of leukemia. The biophysical properties of each leukemia case were extensively compared with physiological cell properties to identify their type and stage. Our cell investigations were compared with results from clinical flow cytometry outcomes. In fact, our method identifies each individual cell type of all the presented leukemia cases and moreover clearly highlights the differences existing between myeloid and lymphoid blasts.

## Results

### Working principle of the detection system

**Figure 1:**
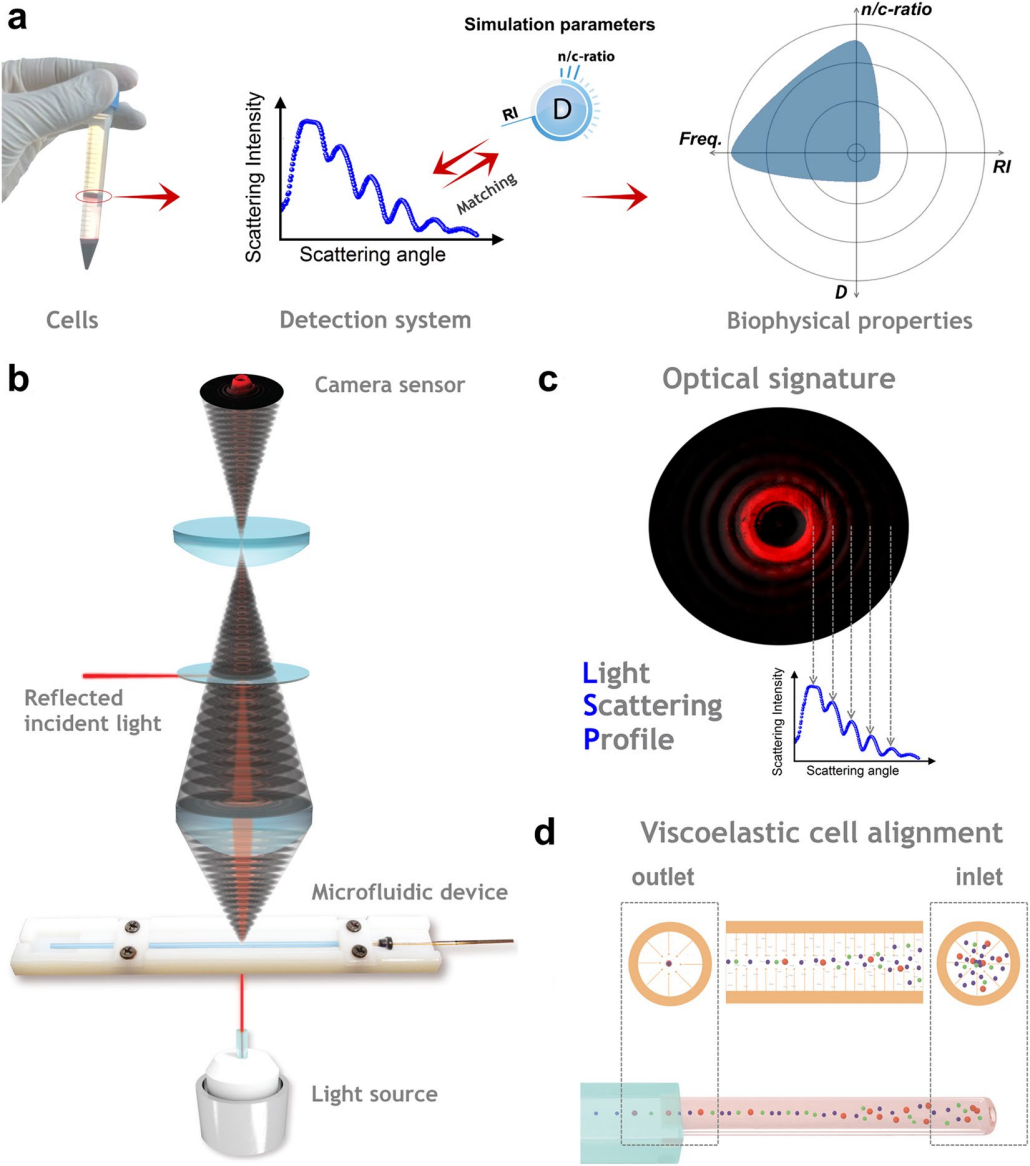
Schematic design of the detection procedure. (**a**) Working principle of the analysis of multiple biophysical properties of a cell, with the following steps: cell collection via density gradient separation; LSP matching with simulation parameters (LSP is calculated from the optical signature); radar plot of biophysical properties from the investigated cells (properties are defined in Fig. 2b, ‘Freq.’ indicates the relative cell frequency). (**b**) Schematic design of the detection system. The incident light source passes the microfluidic alignment stage from the bottom, while the scattered light collection stage maps the obtained optical signature of each passing cell on the sensor (not shown for easier readability). The incident light is reflected out of the signature by a small beam stopper, placed between the optical lenses. (**c**) LSP is calculated out of the signature by averaging the intensity values of all the sensor pixels with same scattering angle. (**d**) The viscoelastic cell alignment over distance in a round capillary is illustrated (flow direction is from the right to the left).

We propose to investigate multiple biophysical properties of unknown cells, by the interaction of an incident light beam with microfluidic aligned cells in sequence, collect its optical signature over a continuous wide scattering range and match the obtained LSP with adequate simulations including the refractive index (RI) of the nucleus, the overall cell dimension D and the n/c-ratio (Fig. 1a).

**Figure 2:**
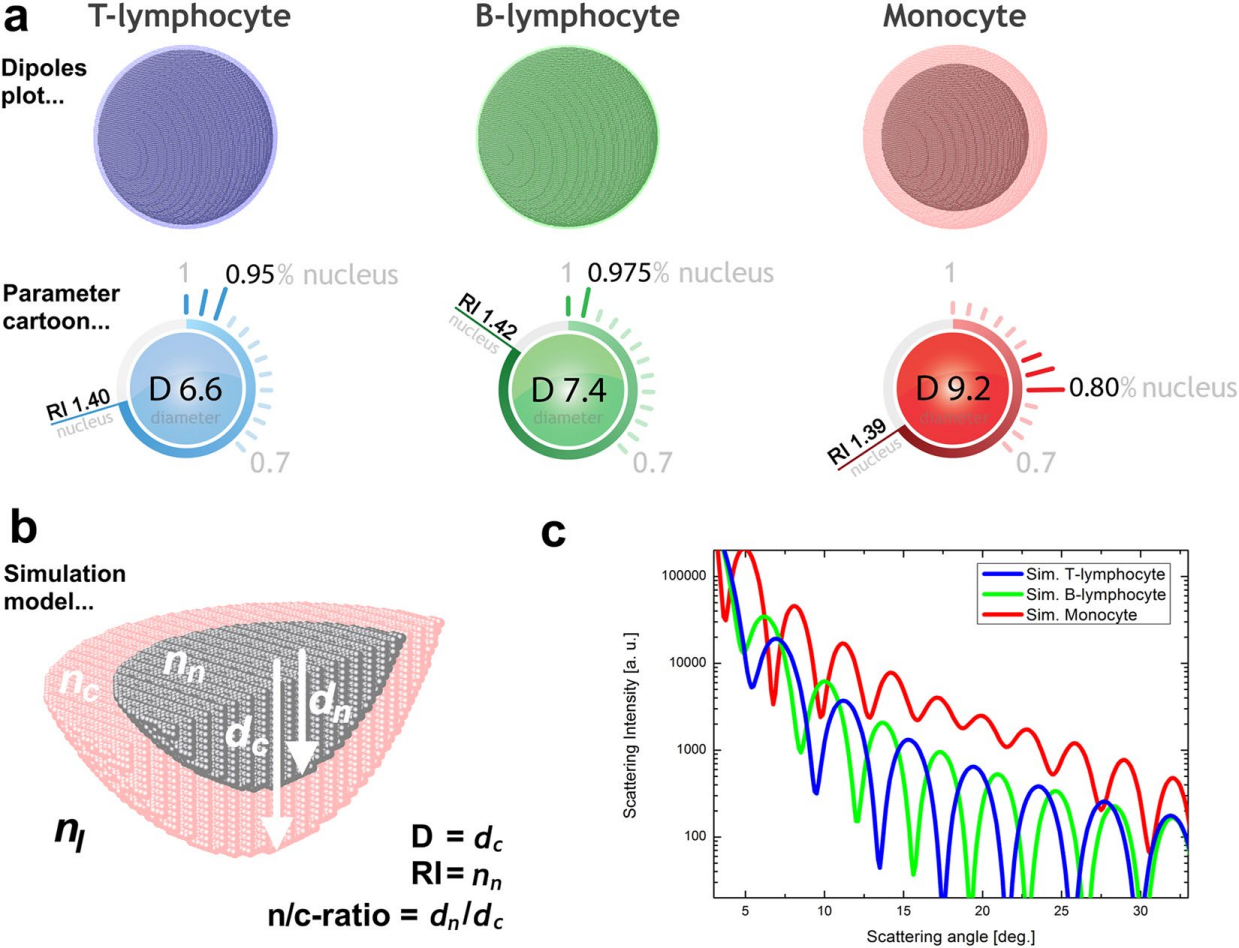
Simulations of PBMC classes with multi-parameter cartoons for direct visualization of the used parameters. Simulation of (**a**) T-lymphocyte, with D = 6.6 µm, RI = 1.40 and n/c-ratio = 0.95; (**b**) B-lymphocyte, with D = 7.4 µm, RI = 1.42 and n/c-ratio = 0.975; (**c**) monocyte, with D = 9.2 µm, RI = 1.39 and n/c-ratio = 0.80 are shown. (**d**) The symmetric simulation model is illustrated; the indices *n*, *c* and *l* are used for nucleus, cytosol, and extracellular liquid, respectively. The nucleus is assumed to be in the center of the cell. (**e**) The LSP simulations for the three presented PBMC classes are plotted together to better visualize the significant LSP differences.

Cells are recovered via simple density gradient separation from fresh whole blood samples. The detection system (Fig. 1b) consists of: an incident laser light source, which passes the microfluidic alignment stage (Fig. 1d) from the bottom; a scattered light collection stage, that directly maps the obtained optical signature of a cell on a sensor; and a data processing software to compute the LSP (Fig. 1c) out of the optical signature. Such obtained LSPs are matched with simulation parameters permitting to obtain significant biophysical properties, which are represented in a radar plot (Fig. 1a).

### LSP simulations of mononuclear blood stream cells

Most scattering techniques in literature are based on the simulation of cells as homogenous spheres with constant properties, while real cells generally present an uneven distribution of biophysical properties, which are strongly related^33^. Moreover, the amount of considered biophysical properties is fundamental to identify cell. In fact, measuring D as a unique property can lead to an incorrect cell interpretation. Therefore, to investigate multiple biophysical properties -including the optical density and dimension of the nucleus (RI and n/c-ratio)- of PBMC cells, a scattering model -based on a coated sphere- was applied to retrieve information from a measured LSP (Fig. 2), which is also reported in literature^34–36^.

A look-up table of possible LSPs was calculated and used to clearly differentiate investigated cells. Each entry of such a table consists of a unique combination of biophysical cell properties and its associated LSP curve, which has been pre-calculated. In fact, using the absolute positions and number of LSP peaks in each simulation curve enabled the unprecedented opportunity to identify unknown cells in-flow (Fig. 2c). Therefore, the cell identification was performed by matching between simulated (look-up table entries) and experimental measured LSP peak positions. LSP minima have not been considered for this analysis. Such a matching procedure can be easily performed by an automatic computer routines in short time.

### System calibration and cell characterization

The presented detection system is very different from the flow cytometry approach, which measures the scattering event of a passing cell at few fixed scattering angles, whereas our approach allows the acquisition of continuous scattering patterns. Moreover, no fluorescence based Ab-labeling or other cell treatments are required. A simple matching of measured LSP with pre-calculated cell simulations permits us to recognize unknown cells by the identification of their unique biophysical properties.

**Figure 3:**
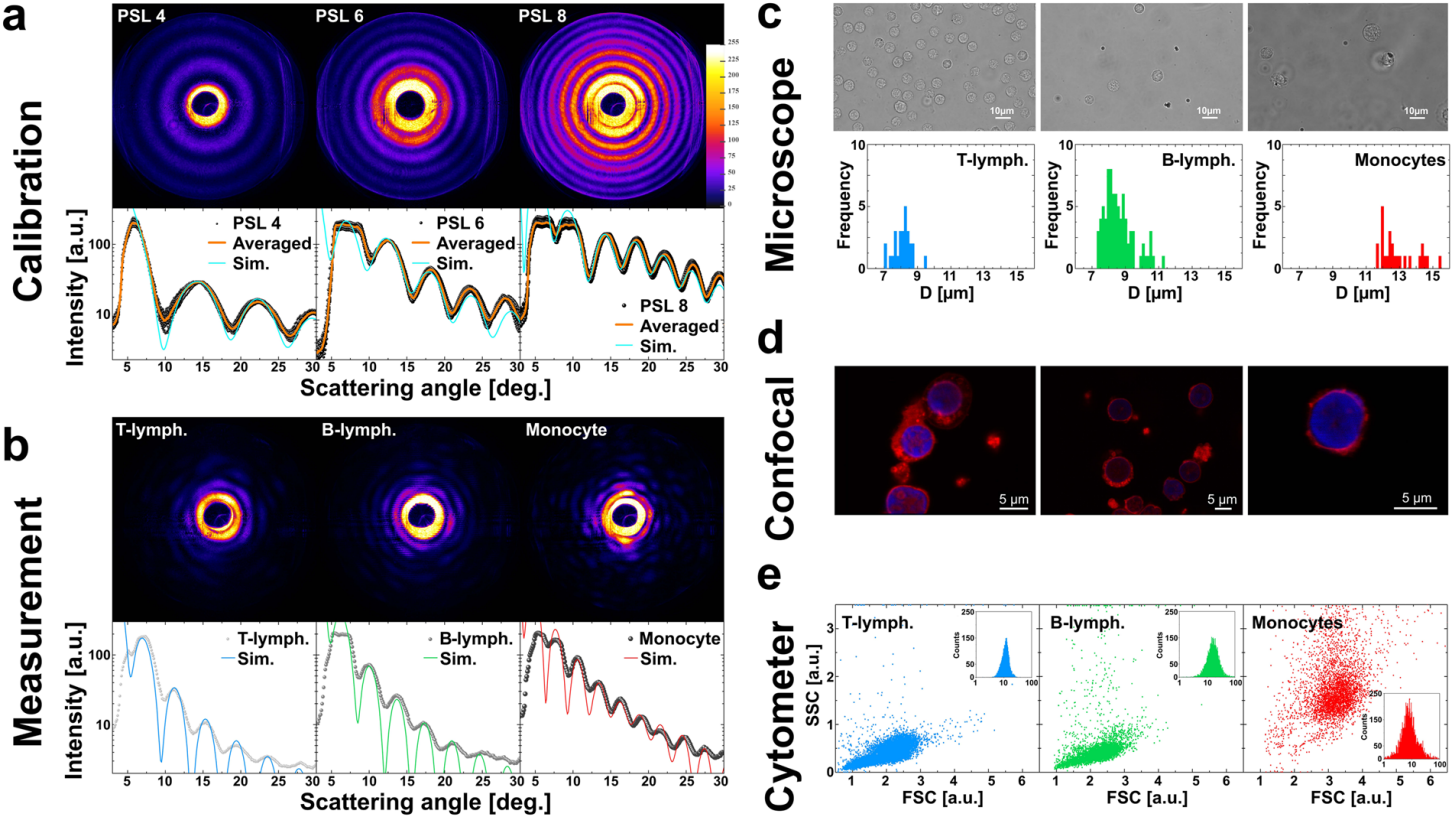
LSPs of PSL particles and PBMC as well as microscope observations. (**a**) On top, typical optical signatures from the Supplementary Video 1 of PSL 4 (38 s), PSL 6 (6 s) and PSL 8 (23 s) are presented (the PSL number indicate the nominal D value). Below LSP outcomes of PSL particles matched with the best fitting simulations are shown (Particle number = 17; RI = 1.587; n/c-ratio = 1.0; D = 4.0, 5.7, 8.0 for PSL 4, 6, 8, respectively). (**b**) A typical LSP and optical signature, obtained from the Supplementary Video 2, of a T-lymphocyte (66 s), B-lymphocyte (80 s) and monocyte (86 s) with overlaid simulation are presented (RI = 1.40, 1.42, 1.39; n/c-ratio = 0.95, 0.975, 0.80; D = 6.6, 7.4, 9.7 for T-, B-lymph. and monocyte respectively). (**c**) Bright field microscope images of each PBMC class (T-, B-lymphocytes and monocytes, from left to right), with histograms of D values obtained by bright field images (outcomes are reported in Supplementary Table 2). (**d**) Confocal images of lymphocytes showing nuclei colored in blue and corresponding cytoplasm in red. No distinction between lymphocyte subclasses are performed. (**e**) Individual separation kits for each presented cell class are tested. For T-lymphocytes, in 11285 events, 99% were detected positive (blue inset). For B-lymphocytes, in 4632 events 98% were detected positive (green inset). For monocytes, in 3262 events, 94% were detected positive (red inset). All reported PBMC classes were isolated by a negative selection procedure using Ab-coupled magnetic beads.

Before characterizing a LSP of a single cell, we must ensure robust alignment conditions for flowing cells. Therefore, we performed calibration measurements of various polystyrene latex (PSL) particle dimensions (Supplementary Video 1). Measurements were performed by diluting different sized monodisperse PSL particles into an appropriate volume of viscoelastic alignment medium and collecting the consequent scattering events (Fig. 3a). We matched the averaged results of PSL measurements with the most adequate simulations, where intensity plateaus, visible at low scattering angles (<12°), were neglected, because they were caused by saturation effects of the camera sensor. The differences between the oscillation peaks of the LSP and the simulation curves were thoroughly investigated in a significant scattering angle range (13°–30°), resulting in a matching percentage error lower than 2%, 3% and 5% for PSL 4, 6 and 8, respectively (Fig. 3a). As result, optimal agreement with manufacture values was found (ID = A, Supplementary Table 1). Moreover, the narrow variance (<1%) of the investigated particle properties can be directly related to a precise and stable 3D-alignment. Since, it is known in literature, that PBMC can be considered as soft particles and, in such a case objects are better aligned compared to rigid ones by viscoelastic forces, the choice of rigid particles for the calibration assure appropriate and robust microfluidic alignment^37^.

Afterwards, we measured physically separated PBMC classes (Fig. 3e) of an adult male donor (ID = D^†^, Supplementary Table 1), obtaining clear and reproducible LSPs (Fig. 3b) and optical signatures for each detected PBMC class, showing matching errors lower than 3% in the scattering angle range of interest (7°–22°). In addition, we investigated each separated PBMC class using standard bright field microscope observations (Fig. 3c), where obtained images were analyzed to derive their D values, neglecting the cell nucleus. Such D values were slightly bigger, but consistent with LSP results (Supplementary Table 2). In addition, PBMC were investigated with a confocal microscope to evaluate the precise relationship between nuclear and cytosolic content of individual cells (Fig. 3d). Results indicated that lymphocytes were mainly occupied by their nucleus. Moreover, we performed the paired-sample t-test on the obtained scattering results and clearly identified independent PBMC classes with p < 0.01 for all measured cell classes.

**Figure 4:**
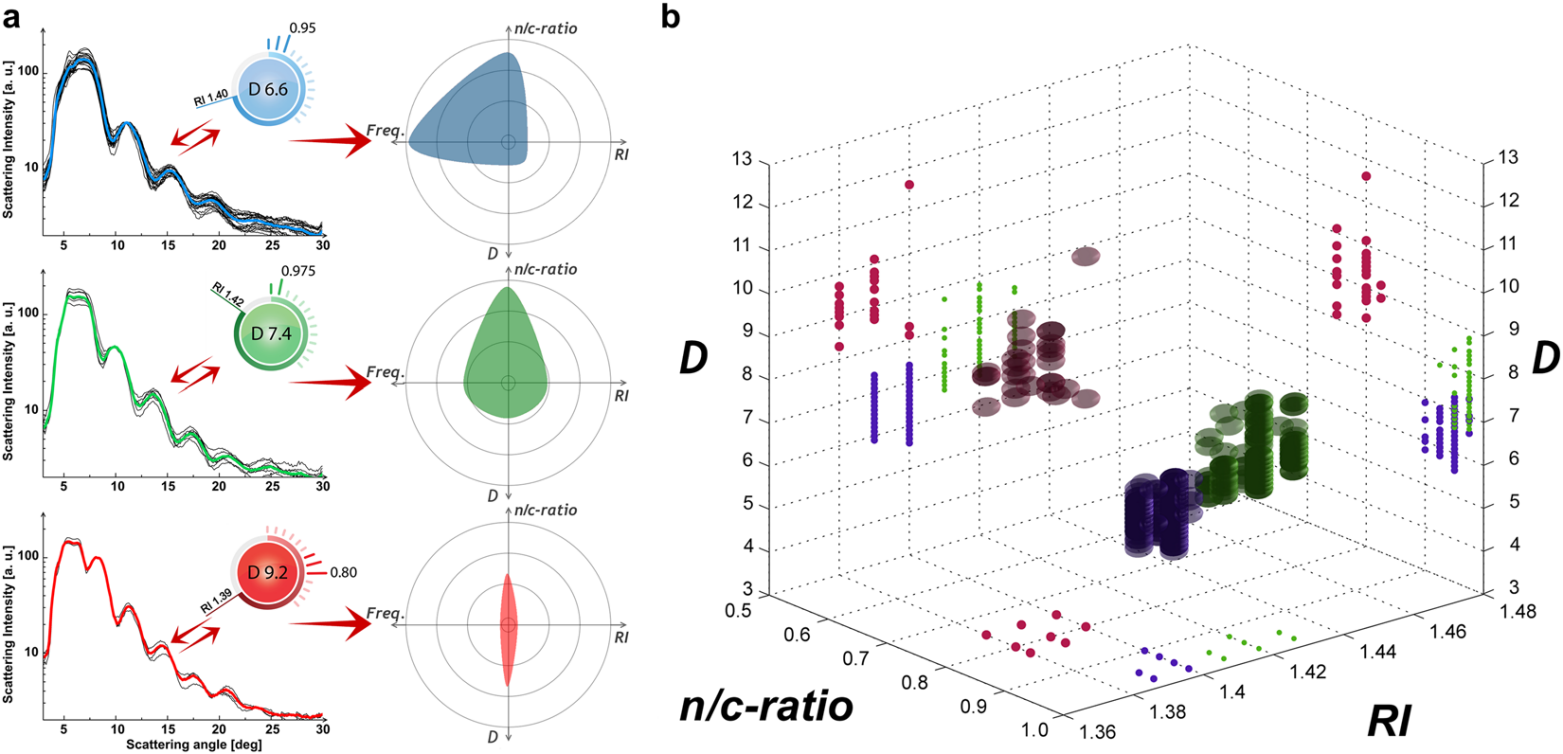
Averaged LSP with best matching parameter cartoons and a multiple properties plot. (**a**) The obtained median biophysical properties (ID = B-E, Supplementary Table 1) are highlighted on the right side for each obtained PBMC class, using blue for T-lymphocytes, green for B-lymphocytes and red for monocytes. (**b**) Multiple properties plot of all measured cells -from individual separated physiological PBMC classes- are reported together (ID = D^†^, Supplementary Table 1). Each class is differently colored for easier readability (blue for T-lymphocytes with 409 cells, green for B-lymphocytes with 204 cells and red for monocytes with 40 cells).

We summarized all PBMC results obtained from individually isolated cell classes, in a multiple properties plot, using the investigated properties as parameter axes (Fig. 4b). We used a different color for each separately measured PBMC class for easier readability, where blue, green and red were used to represent T-, B-lymphocytes and monocytes, respectively. Our detection system is able to appreciate small changes occurring in the biophysical properties of a large population of cells. In other words, we can identify the biophysical characteristics of each single cell by pure LSP measurements, and all three PBMC classes were detected by their distinct biophysical properties. *De facto*, each PBMC class was detected in a different region of the multiple properties plot. Therefore, the number of selected biophysical properties is sufficient to correctly distinguish each PBMC class. Note that, as already mentioned, using D as a unique biophysical property, can lead to an overlapping of the different cell classes and can cause measuring errors.

### Cell measurements from physiological blood samples

We recorded 1,762 PBMCs from different blood samples from healthy male and female donors (ID = B–E, Supplementary Table 1). No cell class isolation by magnetic beads was performed for these samples. LSP were classified by our detection system and the median biophysical properties from each measured cell class was calculated and illustrated as separate radar plot (Fig. 4a). Such median results (Table 1) show significant D value differences for T- and B-lymphocytes -with 6.60 µm and 7.42 µm- while minor changes of n/c-*ratio* −0.95 and 0.975- are detected. RI values of 1.40 for T-lymphocytes and 1.42 for B-lymphocytes were significantly different, giving the opportunity to clearly distinguish them. To our knowledge, we show for the first time in literature a statistically valid distinction between T- and B-lymphocytes by means of pure light scattering measurements^34,38,39^.

**Table 1:** Overview of literature results and experimental measurements for lymphocytes and monocytes.

	Lymphocyte	T-lymphocyte	B-lymphocyte	Monocyte
Brock *et al*.^35^			5.10 µm *n*_*n*_ 1.400; *n*_*c*_ 1.368	
Doowney *et al*.^39^	6.05 µm			8.13 µm
Gascoyne *et al*.^48^		7.00 µm		
Inglis *et al*.^49^	8.50 µm			10.40 µm
Kuse *et al*.^50^	7.30 µm			
Loiko *et al*.^51^	7.50 µm *n* 1.370			9.87 µm *n* 1.370
Polevaya *et al*.^52^		6.80 µm		
Shavlov *et al*.^33^	5.90 µm *n* 1.413			
Strotokov *et al*.^34^		6.38 µm *n*_*n*_ 1.447; *n*_*c*_ 1.376	6.63 µm *n*_*n*_ 1.450; *n*_*c*_ 1.377	
Zheng *et al*.^53^	7.20 µm			
LSP results*		6.60 µm *n*_*n*_ 1.400; *n*_*c*_ 1.360	7.42 µm *n*_*n*_ 1.420; *n*_*c*_ 1.360	9.24 µm *n*_*n*_ 1.393; *n*_*c*_ 1.360

*Averaged values from 1762 cells (ID = B–E, Supplementary Table 1).

We found different biophysical properties of lymphocytes compared to monocytes with D = 9.24 µm, n/c-ratio = 0.794 and RI = 1.39. No significant differences between optical signatures from male and female donors were found. Moreover, PBMC showed similar outcomes compared to the one performed on separated PBMC class investigations.

In a typical PBMC run of 120 s, where 65 scattering events were detected, 32 T-, 14 B-lymphocytes and 1 monocyte were clearly recognized (Supplementary Fig. 1g and Supplementary Video 2). Such cell class frequencies follow the physiologic proportions of PBMC existing in the human blood stream. Note that the relative cell frequency of each cell type is an important information in clinical applications. The presented median biophysical properties of each individual cell class are consistent with literature values. By consideration of D as unique value for the distinction of different cell types, such as T- versus B-lymphocytes, a measurement error up to 60% was detected. Such strong overlapping of an individual cell property implies the need of the combination of several biophysical cell properties to better distinct different cell types. In fact, the superimposition of different cell types was reduced to less than 5% with p < 0.01.

### Cell measurements from leukemia patients

We tested our detection system with pathological (leukemic) cells of eight different children. In a leukemic event, an interruption of the maturation process of blood cell is caused by a genetic aberration occurring in the stem cell. As a result, immature cells multiply and enter in the peripheral vessels^23^. Experiments showed significant changes of biophysical properties and an increased relative frequency of malignant cells compared to their physiological counterparts. Note that PBMC subclass proportions in physiologic conditions can be assumed to be comparable for children and adults^40^.

**Figure 5:**
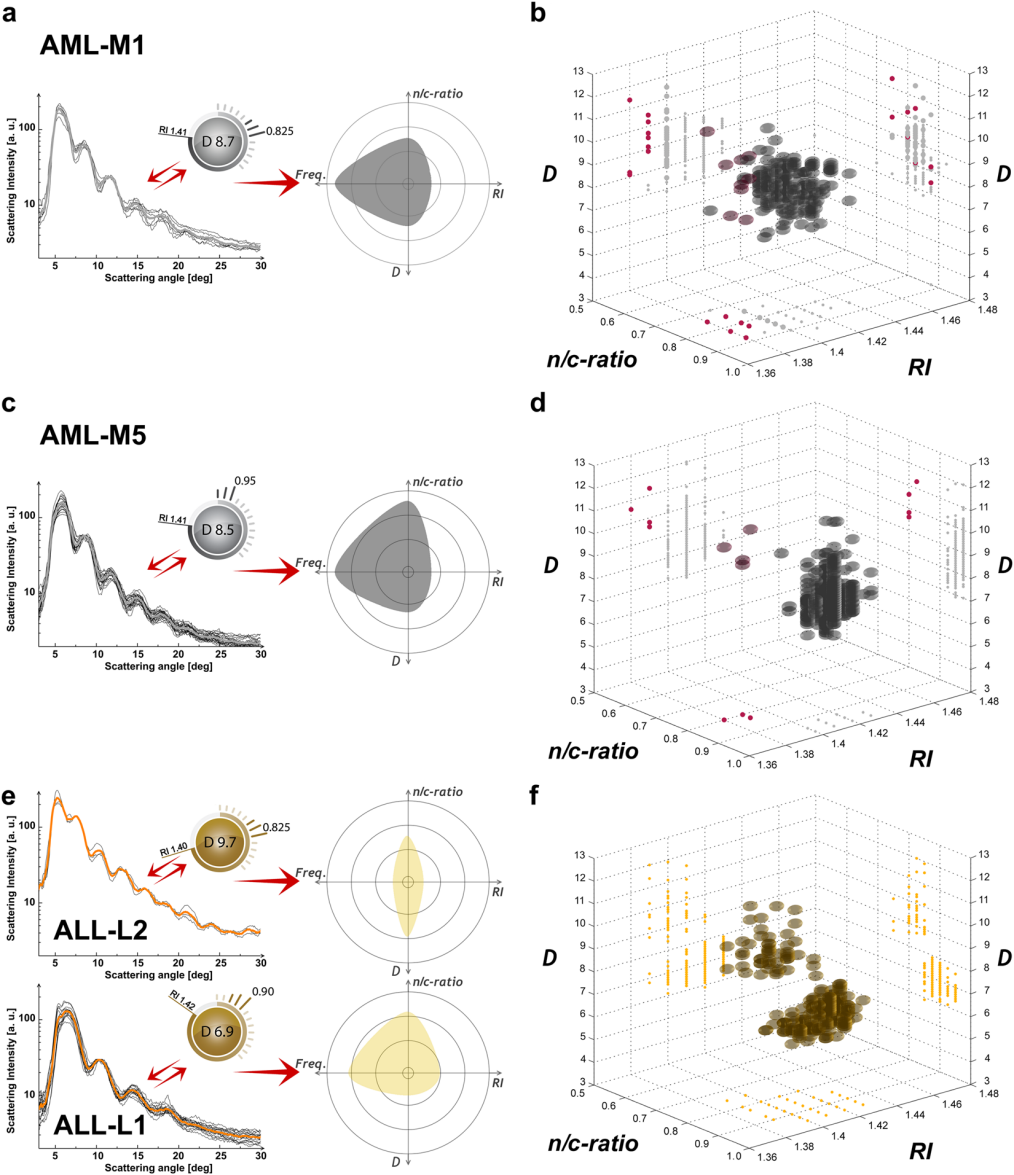
Averaged LSP with matched parameter cartoon for leukemic cells and corresponding multiple properties plots. (**a**) Median biophysical properties of an AML-M1 blood sample are presented (ID = I, Supplementary Table 1). (**b**) Corresponding AML-M1 multiple properties plot. (**c**) Median biophysical properties of an AML-M5 blood sample are presented (ID = G, Supplementary Table 1). (**d**) Corresponding AML-M5 multiple properties plot. (**e**) Median biophysical properties of an ALL blood sample are presented (ID = H, Supplementary Table 1). (**f**) Multiple properties plot of an ALL (ID = H, Supplementary Table 1) blood sample is shown. Each detected cell class is differently colored for easier readability (grey for pathological monocytes with 595 cells, gold for pathological lymphocytes with 167 L1 and 48 L2 cells, red for physiological monocytes).

First, two patients with acute myeloid leukemia (AML) of different pathological cell stages are presented (type AML-M1 in Fig. 5a,b and AML-M5 in Fig. 5c,d). In such rare disease cases, cells show anomalous biophysical properties compared to physiological monocytes: M1 reveal significant bigger D values at higher RI values; M5 cells had significant smaller D values at higher n/c-ratios as well as higher RI values. Our findings were cross-related to microscope observations as well as to flow cytometric analyses, showing consistent results (Supplementary Table 2 and Supplementary Table 3). Beside the pathological cells, a small quantity of physiological monocytes (~1%) was detected in the case of M5 leukemia (red color, Fig. 5d), while in the case of M1 a higher overlapping of biophysical cell properties was detected (Fig. 5b).

In addition, we present an exemplary case of acute lymphoid leukemia (ALL), where B-lymphocytes were affected by biophysical properties changes (more ALL cases are reported in Supplementary Table 1–3 and Supplementary Fig. 1). In general, ALL events can be classified as T- or B-lymphocyte leukemia. In particular, the ALL type affecting B-lymphocytes is the most frequent cancer in children 2–5 years of age^40^. In the presented ALL case, two co-existent different types of leukemic cells were found in one single blood sample (L1 and L2 type). Resulting cells show anomalous biophysical properties: L1 cells had in general D, n/c-ratios and RI values similar to physiological cells, while L2 cells had significantly bigger D values at similar RI and n/c-ratios (Fig. 5e). Whereas the biophysical properties of L1 cells were generally more widespread from the median physiological cell properties, their relative frequency was significantly bigger. In other words, clearly smaller and bigger D values compared to physiological cell observations were detected. Such outcomes were consistent with microscope observations, as well as with flow cytometry analyses. The relative frequency of bigger cell dimensions (L2) was significantly lower, which was consistent with the literature^41^. L1 and L2 showed ~77% and 100% of clearly different results, compared to physiological B-lymphocytes (Fig. 5f). Clinical flow cytometer results show 18% of physiological cells -in the mentioned ALL sample (Supplementary Table 3)- which is consistent with our outcomes, showing 17.86% of physiological cells.

Furthermore, we compared pathologic B-lymphocytes obtained from the peripheral blood with cells directly obtained from the bone marrow of an ALL patient (ID = F & F‡, Supplementary Table 1 and Supplementary Fig. 1a,b). As expected, the investigated cell properties did not show variations between the two blood samples. Moreover, we analyzed pathologic T-lymphocytes of an ALL patient (ID = M, Supplementary Table 1 and Supplementary Fig. 1g). In this case, distinct biophysical properties -mainly affecting cells D values- were detected. Other four leukemic samples of pathologic B-lymphocytes were analyzed with our system showing similar results (Supplementary Table 1 and Supplementary Fig. 1).

Altogether, 2,210 individual optical signatures of different leukemia patients were analyzed and the corresponding biophysical properties of cells were calculated. Small variations -from pathological cells of the same type- were detected, which are natural for a class of patients, whereas most of the pathological cells can be distinguished from the physiological ones. In addition, cells can evolve during the progression of leukemia. However, our screening system investigates cells in short time frames compared to such progressions, allowing to identify different stages of a pathology.

### Physiological versus pathological LSP

**Figure 6:**
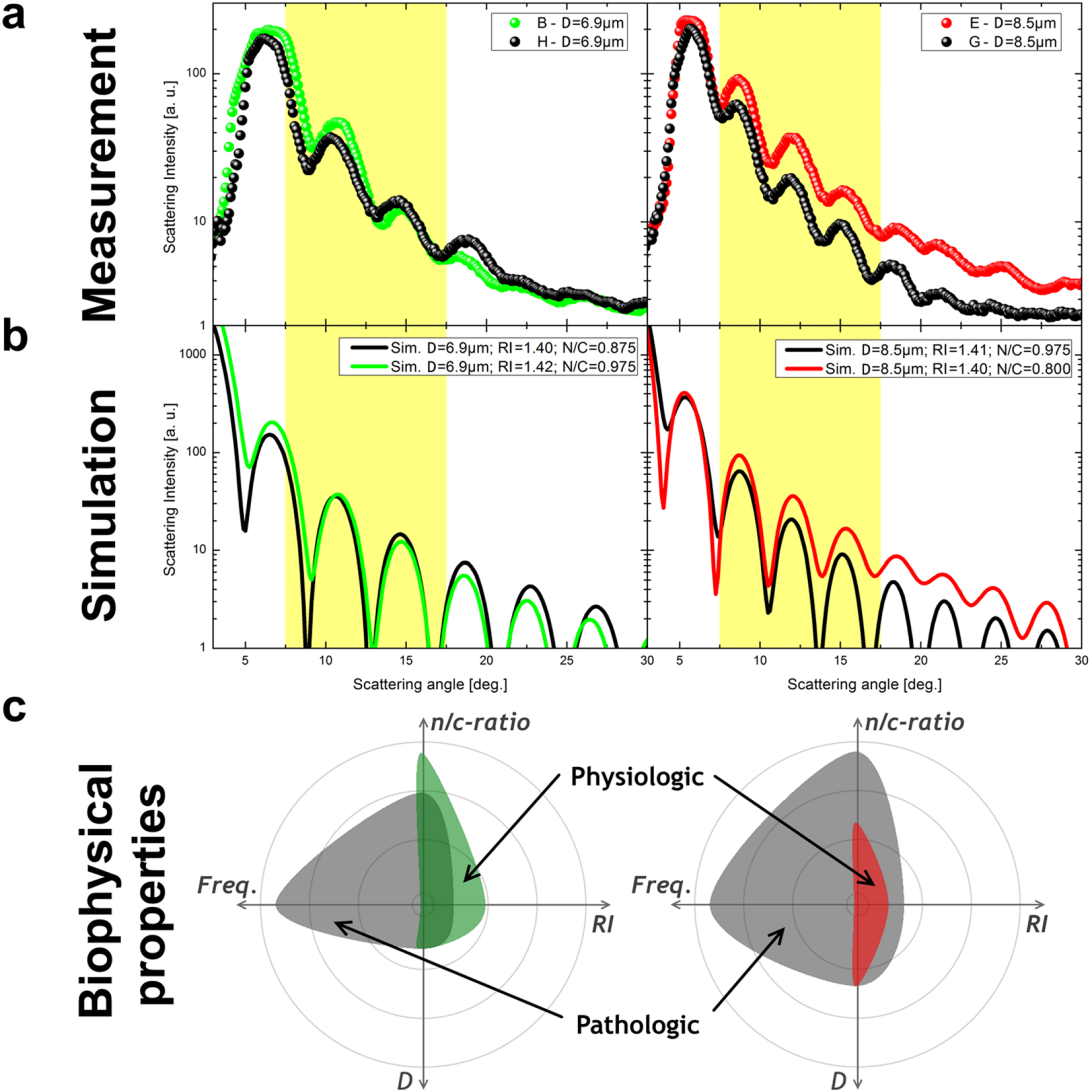
Physiological versus pathological LSPs. (**a**) LSPs for physiological (colored) and pathological (black) cells are presented. The right side shows a case of monocytes, while on the left side lymphocytes are illustrated. (**b**) Corresponding simulation curves are presented. The highlighted yellow scattering angle range of interest clearly shows the differences between physiological and pathological LSPs. (**c**) Radar plots of 1 physiological cell property versus median values of pathological cell measurements are presented. The following parameter ranges are applied: n/c-ratio = 0.7–1.0; RI = 1.38–1.44; D = 6–12; Frequency = 1–1200.

For the identification of different cell stages, it is very common to mainly refer to D value, which fails in the case of leukemia. We show the clear need of multiple biophysical properties for an adequate label-free cell stage identification. Therefore, we plotted a typical physiological lymphocyte as well as a monocyte versus the corresponding pathological cells only considering their D values (Fig. 6a). The shifting between the two curves in the highlighted scattering angle range of interest (7.5°–17.5°) are in general not considered by standard light scattering investigations. To emphasize our findings, we also plotted the corresponding simulation curves below (Fig. 6b), which showed the same LSP trend. These LSP variations between physiological and pathological cells of same D value clearly indicate the need of a multiple properties approach to correctly interpret label-free light scattering results. The observed changes in LSPs are related to the oscillation slope and its absolute scattering peak intensities. Beside such LSP changes the plotting of biophysical properties in a radar plot clearly indicates distinct differences of an individual physiological versus a class of pathological cells (Fig. 6c). Such a radar plot report that, for constant D values, pathological cells reveal significantly different RI values as well as n/c-ratios, compared to physiological ones. In doing so we could underline the different biophysical properties of individual physiological versus pathological cells of same overall dimension.

In addition, for a more profound distinction between different cell classes and/or stages, we have performed cluster analysis of biophysical properties obtained for each detected cell type. First we have analyzed PBMC outcomes from an individual donor (ID = D), using a paired-sample t-test approach to verify that all detected cell classes (T-, B-lymphocytes and monocytes) are distinct and normally distributed. On top of that, we performed a cluster analysis, using the k-means clustering partitions approach. In doing so, 2% of PBMC were detected differently compared to our outcomes (Supplementary Table 4 & Supplementary Fig. 2). Second, we have analyzed, with the previously mentioned statistical approach, pathological versus physiological outcomes from all analyzed patients (ID = F-M), showing mainly similar results (Supplementary Table 4). In some cases (ID = F, F^†^ and L) a high superimposition was detected. Such an outcome implies that the detected cells have biophysical cell properties, which are similar to the one of physiological B-lymphocytes. Nevertheless, the fact that no monocytes or T-lymphocytes were detected in this blood sample can be sufficient for the clinicians to start further investigations or even patient treatment. For the case of a patient with myeloid pathology of early stage M1 (ID = I), a high percentage of analyzed cells were detected with biophysical cell properties similar (n/c-ratio = 0.825) to the one of physiological monocytes (n/c-ratio = 0.800). This result is expected, due to fact that such cells are less different from physiological ones compared to more advanced pathological stages (n/c-ratio = 0.950), M5 (ID = G).

## Discussion

We presented a single-cell screening approach based on the label-free measurement of multiple biophysical properties in microfluidic flow, only using their individual optical signatures. Out of each signature we calculated a scattering profile, which we matched with the best fitting simulation to retrieve a set of intrinsic cell properties (D, n/c-ratio, RI). We demonstrated, that it was possible to identify cell classes and stages, from such precise single cell properties investigations. The high potential of our detection system lies in the appropriate combination of the most significant biophysical cell properties, which permit fast and precise characterization of unknown cells. Experimental measurement conditions have been set to assure maximum surviving and morphological preservation of the analyzed cells. Our method can identify more than 1,000 individual cells and their belonging to different physiological or pathological types in measurement runs lasting less than 1 hour. Improved cell throughput rates -up to 50,000 individual cells per hour- could be achieved using smaller measurement channel cross-sections and/or higher applied *∆P*-values. Nevertheless, an ordinary morphometric cell shape is not ensured for such high throughput rates.

Here, we reported a detailed study of physiological PBMC, that permitted us to obtain a precise and distinctive classification of T-, B-lymphocytes and monocytes. In particular, for T-, B-lymphocytes and monocytes median D values of 6.60 µm, 7.42 µm and 9.24 µm, were detected. The second biophysical property considered for our classification is the nucleus-to-cytosol ratio, which was found to be close to 1 for T- and B-lymphocytes, (0.950 and 0.975 respectively) and significantly lower for monocytes (0.794). Moreover, RI as the third investigated biophysical property showed a median value of 1.39 for monocytes, while values for T- and B-lymphocytes were significantly higher, 1.40 and 1.42, respectively. Furthermore, the presented single-cell analysis allows us to quantify the number of cells with equal biophysical properties, expressed by the relative cell frequency. In fact, for each measurement, we statistically recovered the natural PBMC cell class occurrence. Therefore, by adequate combination of the presented biophysical cell properties, it is possible to distinguish PBMC classes. As results, for the first time in literature a functional light scattering-based discrimination of T- and B-lymphocytes, without recurring to any labelling approach, was presented.

We tested our approach with pathological blood samples of child leukemia, showing substantial biophysical properties differences between pathological and physiological cells. Most leukemic cells presented anomalous values of at least two of the mentioned biophysical properties, permitting a fast and reliable identification of different stages of lymphoid and myeloid leukemia cell differentiation. The specific kind of leukemia (myeloid or lymphoid) and its relative stage (M1 or M5; L1 or L2) was detected by our label-free screening system. In particular, distinct changes of the biophysical cell properties strictly correspond to state of the art outcomes and, therefore, to the specific clinical pathology. Beside the possibility of a clear distinction between pathological or physiological cells, by means of reported biophysical measurements, the relative cell frequency is also a fundamental parameter for the diagnosis and prognosis of leukemia. In summary it can be stated, that in any investigated blood sample our method showed a perfect matching with clinical flow cytometer outcomes. Moreover, we found increasing nucleus sizes for leukemic cells, with generally increased overall cell dimensions, which is in good agreement with conventional measurement results. Nevertheless, an extended study on more patient samples could validate the importance of such an approach in the clinical field.

Our method is straightforward, inexpensive, not requiring any costly cell-labeling or complex cell preparation steps. In general, it is possible to investigate any kind of cell -even rare cells- retrieving its biophysical properties by matching measured optical signature with the appropriate theoretical scattering model. Such a potentiality is of great interest when very few pathological cells can have a significant diagnostic impact (minimal residual disease, circulating tumor cells). We believe that our approach could represent a useful tool for a label-free analysis of biophysical single-cell signatures.

## Materials and Methods

### Peripheral blood mononuclear cell recovery

A minimum of 12 mL of human blood samples from healthy adult donors or circa 3 ml of leukemia child patients were withdrawn with a standard venepuncture procedure. All samples were taken after obtaining informed consent from all donor, or their legal guardians in accordance with relevant guidelines and regulations. The experimental protocol was approved by a licensing committee (EU clinical trial register - EudraCT Number: 2007-004270-43). All patients gave informed consent to publish identifying information. All blood samples were stored in standard K_2_EDTA tubes (Vacutainer, BD) to prevent coagulation, and measured within 1 hour from collection. By using a density gradient centrifugation procedure, PBMC were obtained from the samples. Because platelet contamination is very common on such separations^42^, specific adjustments were made to the standard separation procedure to get rid of the unwanted platelets which could interfere with scattering measurements^43^.

Each blood sample was diluted 1:1 with a pure phosphate-buffered saline (PBS, EUROCLONE), laid on the top of an equal volume of density media (Histopaque-1077, SIGMA ALDRICH) and centrifuged at 300 $\overrightarrow{g}$ for 25 min (deactivated centrifuge brake). The resulting PBMC ring was collected and washed twice with 10 mL of red blood cell lysis solution to eliminate contaminating erythrocytes.

### Cell isolation and validation

PBMC classes were isolated by a negative selection procedure using Ab-coupled magnetic beads (Dynabeads Untouched kit, INVITROGEN). After PBMC recovery from whole blood, samples were incubated with the magnetic beads and coupled with Ab binding all the unwanted cell classes. The unwanted cells, covalently bound to magnetic particles, were retained to the tube surfaces thanks to a magnet and finally, the desired cell class was recovered untouched. Isolation purity was assessed using a commercial flow cytometer (CyFlow SPACE, SYSMEX-PARTEC) with the corresponding Ab (SYSMEX-PARTEC) for cell identification: anti CD3-FITC for T-lymphocytes, anti CD19-PE for B-lymphocytes or anti CD14-PE for monocytes (Fig. 3e).

Leukemic cell samples, on the contrary, did not require any specific isolation steps. Cells were directly obtained from the whole PBMC population and suspended in the microfluidic alignment solution, ready for successive tests.

To perform correct cell investigations in-flow, physiological cell-like surrounding conditions are essential. For such a reason, right before and after each scattering measurement, detailed visual morphometric cell observations -using the viscoelastic alignment medium- were obtained. Such vitality tests were performed using the ‘Trypan blue exclusion test’, where no considerable morphological cell modifications or mortality were observed. A standard bright-field microscope (X81, OLYMPUS) with a 100X oil immersion objective was used to obtain D values of PBMC, while reference values for nucleus over cytosol ratios investigations were observed with a confocal microscope (TCS SP5 II, LEICA) using a 100X oil immersion objective, with alternating additional magnifications. Hereby the average dimension of the nucleus was divided by the average dimension of the whole cell. For confocal observation cells were bound to a glass surface plate thanks to the use of a poly-L-lysine coating. Subsequently, cells were stained with a fluorescent nuclear colorant (Hoechst 33342, SIGMA ALDRICH) and a cytosolic one (CellTracker Red CMTPX Dye, THERMO FISHER) and fixed using paraformaldehyde at 4%.

### Viscoelastic cell alignment

Our research group has recently shown the possibility to align erythrocytes in sequence -by viscoelastic forces- in a cost-effective and simple way^18^ and further developed this alignment approach for PBMC investigations. Hereby a pressure-driven viscoelastic solution -consisting of 0.2 g of polyethylene oxide (PEO − M_w_ = 4 MDa, SIGMA-ALDRICH) diluted in 1 dL of pure PBS- aligns cells or micrometric particles in a round shaped capillary thanks to its elastic properties, before passing in a subsequent square shaped channel, where cells are investigated at preserved alignment conditions. The alignment probability^44^ to the centerline of the capillary can be expressed by a dimensionless parameter *θ*,

1 $$\theta =\gamma ^{\prime} {\lambda }_{t}+{\beta }^{2}(L/{r}_{c})$$

with the relaxation time *λ*_*t*_ = 0.123 *ms* of the polymer solution and geometric parameters such as the confinement ratio *β*

2 $$\beta ={d}_{c}/2{r}_{c}.$$

Moreover, the capillary length *L* = 0.3 *m* and the average shear rate *γ*′ of 769 *sec* ^*−1*^ of the polymer solution are relevant for the alignment probability, where *γ*′ is defined as

3 $$\gamma ^{\prime} =({\rm{\Delta }}P{r}_{c})/(8{\eta }_{0}L),$$

with ∆*P* (1000 *mbar*) defining the applied pressure (P-pump, DOLOMITE) to push the sample through the capillary and *η*_0_ = 0.0054 *pas* the zero-shear viscosity of the polymer solution.

In conclusion, a sufficient alignment condition exceeding *θ* ≥ 1 can be simply achieved by an appropriate setting of all the previously mentioned cell alignment parameters. At the end of the round shaped capillary a *θ* ranging from 34 until 111 was achieved for *d*_*c*_ values of 5 to 9 *µm*, to achieve the required precise 3D-alignment of cells in-flow.

All scattering measurements were performed with a flow rate of ~100 µm/s through the square capillary of the microfluidic device, to ensure an accurate cell analysis in sequence. We suspended cells in a highly diluted viscoelastic alignment medium to reach a final concentration of ~1,000,000 cells/ml. Thus, a variable living cell detection throughput up to 1.2 cells/s could be obtained. Nevertheless, such a microfluidic cell environment ensured that the investigated cells pass the light beam alive and without any considerable physical variations at a constant 3D-alignment position. Moreover, it permitted the cells to survive the whole measurement procedure without addition of substances designed for cell nourishment -like glucose- and allowed subsequent cell investigations. In such a way, the alignment capability was totally preserved, and no unwanted scattering events from the alignment medium occurred during the measurements.

### Modeling of mononuclear blood stream cells

We used a coated sphere model, based on the discrete dipole approximation (DDA) method, to simulate theoretical scattering responses of cells. This volume-based simulation method approximates the cell by a lattice of dipoles, where the number of dipoles for each simulation strongly depends on D. Around 2.2 × 10^6^ dipoles (core: 1.6 × 10^6^, shell: 0.6 × 10^6^) placed in a 162 × 162 × 162 grid were computed for a typical PBMC. We used the free available ADDA source code (v.1.3b4)^45^ performed on a personal computer for all our DDA simulations (Fig. 2a). Such a simulation model assumes cells to be in suspension, while for standard histological observations cells tend to be stained on a glass slide.

We calculated a look-up table of more than 20,000 entries with the following PBMC parameters: D values from 4–12 µm (step size = 0.1 µm), n/c-ratio from 0.5–1.0 (step size = 0.25) and RI of the nucleus from 1.38–1.48 (step size = 0.01), at a constant RI of the cytoplasm equal to 1.36. We related RI to the optical density of nucleus or cytoplasm. All simulation parameters were defined as shown before (Fig. 2b) and chosen according to common literature values^34,36,46^.

### Detection system

The presented camera based label-free detection system consists of: an incident laser light source, a scattered light collection stage and a data processing software (Fig. 1b). It allowed us to acquire a continuous LSP of single particles or cells in a scattering range of 3°–30°, with an optical resolution of 0.1022°. The recorded scattering images were generated by the interaction of a narrow-collimated incident laser beam (wavelength of 632.8 nm) -passing a highly transparent microfluidic channel from the bottom- and a viscoelastic aligned individual particle or cell while flowing in our microfluidic system with submicron range resolution. The basic working principle and used optical equipment are reported elsewhere^47^ in more detail.

During the scattered light collection stage (Fig. 1b), the incident light is reflected to the side by a beam stopper (blocking scattering angles <3°), while the resulting optical signature of a passing object is collected by a camera sensor for the LSP calculation. Thereby, each scattered light speckle of same magnitude of the scattering wave-vector, but with different azimuthal orientations, is collected in a single scattering ring. The average intensity of each ring is combined to the continuous LSP over the obtained scattering range (Fig. 1c). In such a simple straightforward measurement procedure, a microfluidic based multiple biophysical readout of the investigated objects gets possible. The cell alignment stage consists of a microfluidic device and a commercially available pressure pump. The new designed cost-effective microfluidic device use a base, produced by a 3D printer (Objet30 Pro, STRATASYS), one square (VitroTubes, VITROCOM) as well as one round (flexible fused silica capillary tubing, POLYMICRO TECHNOLOGIES) capillary, a soft ferrule (NanoPort Ferrules, IDEX HEALTH & SCIENCE) and 4 screws. The base is used to fix in place a commercially available hollow square capillary of 500 × 500 µm and beyond that is designed to let the full amount of incident laser beam pass without interfering the scattering process. In addition, the base is used to locate a soft ferrule at the entrance of the square capillary (Fig. 1b). Thereby, the squared capillary entrance is fully sealed by the ferrule and a round shaped capillary with an inner diameter of 50 µm and outer diameter of 363 µm can pass the ferrule from the opposite side until the entrance of the squared capillary. The other side of the round capillary is immersed in the measurement sample and a certain pressure is applied to push the sample through the microfluidic device. In such a way, we simply and cost-effectively connect a round with a square capillary. Note that the centerlines of both capillaries are placed collinear for optimal cell alignment conditions (Fig. 1d).

### Percentage error calculation

To validate the matching procedure of the detection system between experimental and simulated LSP curves, the percentage error of the matching is calculated. In such a case the intensity values of LSP peaks in a certain significant scattering range of interest are compared, resulting in less than 3% and 5% of matching error for investigated cells and particles, respectively (Supplementary Table 1). LSP with higher percentage error values are automatically excluded by the matching routine.

### Cluster analysis

To validate the existence of distinct differences between the detected biophysical cell properties for each analyzed cell type, we performed a statistical analyzes of our outcomes, using the k-means clustering partitions approach. Hereby k describe the mutually exclusive clusters of the analyzed data. In general, these techniques assign each cell obtained from an optical signature (biophysical cell properties – D, n/c-ratio and RI) to a specific cluster by minimizing the distance from the data point to the mean location of its assigned cluster. For all calculations, k was chosen according to the expected cell type number in the analyzed blood sample. In the case of pathological blood samples, a certain number of physiological data was added to the input data, to better investigate their differences to physiological cells (Supplementary Table 4, Supplementary Figs 2–4).

## Electronic supplementary material

Supplementary Info

Supplementary Video 1

Supplementary Video 2
