# Supplementary material for: Single-cell screening of multiple biophysical properties in leukemia diagnosis from peripheral blood by pure light scattering
Source: Sci Rep. 2017 Oct 4;7:12666. doi: 10.1038/s41598-017-12990-4 (PMC5627307; doi:10.1038/s41598-017-12990-4)
Supplement: Supplementary file 1 — Supplementary Info [file 41598_2017_12990_MOESM1_ESM.pdf]

Supplementary info:

Single-cell screening of multiple biophysical properties in leukemia diagnosis from peripheral blood by pure light scattering

David Dannhauser[1,*], Domenico Rossi[1], Mimmo Ripaldi[2], Paolo A. Netti[1,3] & Filippo Causa[3,*]

[1] Center for Advanced Biomaterials for Healthcare@CRIB, Istituto Italiano di Tecnologia (IIT), Largo Barsanti e Matteucci 53, 80125 Naples, Italy.
[2] BMT Unit, Department of Pediatric Hemato-Oncology, Santobono-Pausilipon Hospital, Via Posillipo, 226, 80123 Naples, Italy.
[3] Interdisciplinary Research Centre on Biomaterials (CRIB) and Dipartimento di Ingegneria Chimica, dei Materiali e della Produzione Industriale, Università degli Studi di Napoli "Federico II", Piazzale Tecchio 80, 80125 Naples, Italy.
*Correspondence to [david.dannhauser@iit.it, causa@unina.it]

Supplementary Video1. The video shows optical signature measurements of a mixed PSL sample (PSL 4, 6, 8) recorded by the camera sensor of the detection system. In general, the higher the obtained scattering intensity the brighter the pixel color appears, while the central area of the video remains dark caused by the beam stopper, reflecting the incident light out of the optical signatures. The higher the number of obtained intensity rings in an obtained optical signature, the bigger the obtained PSL dimension and *vice versa*. Three different signature types can be recognized in this video, according to the mixed particle dimensions reported in supplementary Table 1.

Supplementary Video2. The video shows optical signature measurements of a PBMC blood sample (T-lymphocytes, B-lymphocytes and monocytes) of 120s, recorded by the camera sensor of the detection system. Out of such records signatures of each individual cell, the LSP are calculated and subsequently the multiple biophysical properties are detected. The higher the obtained intensity the brighter the pixel color appears. The central area of the video remains dark caused by the beam stopper, reflecting the incident light out of the optical signatures. This video shows less symmetric optical signatures compared to supplementary Video 1, caused by the natural variety of the cell shapes. The higher the number of intensity rings the bigger a cell dimension can be assumed. For more detailed analysis, the LSP of each scattering event is calculated by a measurement routine and used for the matching with the best fitting pre-calculated simulations of possible cell properties.

Supplementary Table 1. Multiple biophysical property investigations of all analyzed particles and cells. Physiological lymphocytes and monocytes were detected out of pure PBMC samples if not otherwise indicated. The ID = B-E indicate the summarized average values for the donors B, C, D and E. Nominal values of PSL 4, 6 and 8 particles (SIGMA ALDRICH) are 4.16±*0.06* µm, 6.08±*0.08*µm and 8.02±*0.10* µm, respectively.

ID	*Type*	*Gender*	*n/c-ratio**	*RI**	*D [µm]*	*Number*	*Matching Error [%]*
A	PSL 4	-	1.000	1.59	4.00 ±*0.10*	17	1.9
A	PSL 6	-	1.000	1.59	5.70 ±*0.11*	17	2.7
A	PSL 8	-	1.000	1.59	7.95 ±*0.11*	17	4.7
B	PBMC - T	Female	0.950	1.40	6.63 ±*0.34*	402	1.1
C	PBMC - T	Female	0.950	1.40	6.32 ±*0.31*	104	1.1
D	PBMC - T	Male	0.950	1.40	6.75 ±*0.35*	463	1.3
E	PBMC - T	Male	0.950	1.40	6.69 ±*0.43*	193	1.7
B-E	PBMC - T	Mixed	0.950	1.40	6.60 ±*0.36*	1162	1.3
B	PBMC - B	Female	0.975	1.42	7.31 ±*0.47*	207	1.3
C	PBMC - B	Female	0.975	1.42	7.45 ±*0.57*	53	1.1
D	PBMC - B	Male	0.975	1.42	7.34 ±*0.48*	123	1.2
E	PBMC - B	Male	0.975	1.42	7.57 ±*0.51*	135	1.7
B-E	PBMC - B	Mixed	0.975	1.42	7.42 ±*0.51*	518	1.3
B	PBMC - M	Female	0.800	1.39	9.29 ±*0.71*	28	2.4
C	PBMC - M	Female	0.800	1.39	9.33 ±*0.83*	12	1.7
D	PBMC - M	Male	0.750	1.39	8.96 ±*0.72*	16	1.2
E	PBMC - M	Male	0.825	1.40	9.40 ±*0.44*	26	2.6
B-E	PBMC - M	Mixed	0.794	1.39	9.24 ±*0.67*	82	2.0
D†	PBMC - T	Male	0.950	1.40	6.78 ±*0.35*	409	1.2
D†	PBMC - B	Male	0.975	1.42	7.15 ±*0.56*	204	1.5
D†	PBMC - M	Male	0.800	1.39	9.12 ±*0.64*	40	2.2
F	ALL-L1 (type B)	Female	0.975	1.42	7.50 ±*0.87*	220	1.8
F‡	ALL-L1 (type B)	Female	0.975	1.43	7.20 ±*0.81*	165	1.7
G	AML-M5	Female	0.950	1.41	8.45 ±*0.69*	595	1.3
H	ALL-L1 (type B)	Male	0.900	1.42	6.94 ±*0.40*	167	2.5
H	ALL-L2 (type B)	Male	0.825	1.40	9.70 ±*0.87*	48	2.1
I	AML-M1	Female	0.825	1.41	8.67 ±*0.82*	179	1.4
J	ALL-L2 (type B)	Male	0.950	1.42	7.90 ±*0.78*	162	1.0
K	ALL-L1 (type B)	Male	0.925	1.42	8.30 ±*1.01*	142	1.1
L	ALL-L1 (type B)	Female	0.975	1.42	6.84 ±*0.99*	252	2.4
M	ALL-L1 (type T)	Female	0.950	1.40	9.07 ±*1.21*	280	2.4

* Median values, all standard deviations are below 3.6%.
† Cell class separation kit has been used.
‡ Blood sample from the bone marrow.

Supplementary Table 2. Bright filed microscope results from separated PBMC classes of donor D and pathological cells from leukemic blood samples.

ID	*Type*	*D [µm]*	*Number*
D	PBMC - T	8.01 ±0.59	29
D	PBMC - B	8.60 ±0.88	81
D	PBMC - M	12.82 ±1.35	29
F	ALL-L1 (type B)	9.31 ±1.09	30
F‡	ALL-L1 (type B)	9.44 ±1.04	27
G	AML-M5	9.16 ±0.71	22
H	ALL-L1 (type B)	11.60 ±1.10	18
H	ALL-L2 (type B)	8.33 ±0.86	18
I	AML-M1	10.25 ±1.11	23
J	ALL-L2 (type B)	8.62 ±0.88	27
K	ALL-L1 (type B)	9.55 ±0.92	24
L	ALL-L1 (type B)	8.03 ±1.06	26
M	ALL-L1 (type T)	10.70 ±1.40	21

‡ Blood sample from the bone marrow.

Supplementary Table 3. Clinical flow cytometer based leukemic blood samples analyzes by standard Ab-labelling technique.

ID	*Analyzed CDs*	*Lymphoid*	*Myeloid*	*Cytometer results*	*Final Diagno sis*
F	CD45, CD2, CD3, CD5, CD7, CD10, CD11a, CD11b, CD11c, CD13, CD14, CD15, CD16, CD19, CD20, CD33, CD34, CD38, CD56, CD58, CD64, CD99, CD117, HLA-DR, TdT, MPO	Nearly whole (13% of physiological lymphocytes)		CD45, CD19, CD10, CD38, HLA-DR, CD34, TdT	ALL common
G	CD45, CD2, CD3, CD5, CD7, CD10, CD11a, CD11b, CD11c, CD13, CD14, CD15, CD16, CD19, CD20, CD33, CD34, CD38, CD56, CD58, CD64, CD99, CD117, HLA-DR, TdT, MPO	4%	90%	(Myeloid) CD45dim; CD33, CD13, CD34, CD117, CD38, HLA-DR, TdT	AML
H	CD45, CD2, CD3, CD5, CD7, CD10, CD11a, CD11b, CD11c, CD13, CD14, CD15, CD16, CD19, CD20, CD33, CD34, CD38, CD56, CD58, CD64, CD99, CD117, HLA-DR, TdT, MPO	Nearly whole (18% of physiological lymphocytes)		CD45, CD19, CD10, CD38, CD34, HLA-DR, TdT	ALL common
I	CD45, CD2, CD3, CD5, CD7, CD10, CD11a, CD11b, CD11c, CD13, CD14, CD15, CD16, CD19, CD20, CD33, CD34, CD38, CD56, CD58, CD64, CD99, CD117, HLA-DR, TdT, MPO	3%	90%	(Myeloid) CD45, CD7, CD33, CD13, CD34, HLA-DR, MPO, CD15+-	AML
J	CD45, CD2, CD3, CD5, CD7, CD10, CD11a, CD11b, CD11c, CD13, CD14, CD15, CD16, CD19, CD20, CD33, CD34, CD38, CD56, CD58, CD64, CD99, CD117, HLA-DR, TdT, MPO		Nearly whole	CD45, CD19, CD38, HLA-DR, CD15+-, CD34, MPO+-, CD10-	ALL pro-B
K	CD45, CD2, CD3, CD5, CD7, CD10, CD11a, CD11b, CD11c, CD13, CD14, CD15, CD16, CD19, CD20, CD33, CD34, CD38, CD56, CD58, CD64, CD99, CD117, HLA-DR, TdT, MPO	Nearly whole (10% of physiological lymphocytes)		CD45, CD19, CD10, CD38, HLA-DR, CD34+-, CD20+-	ALL common
L	CD45, CD2, CD3, CD5, CD7, CD10, CD11a, CD11b, CD11c, CD13, CD14, CD15, CD16, CD19, CD20, CD33, CD34, CD38, CD56, CD58, CD64, CD99, CD117, HLA-DR, TdT, MPO	Nearly whole (8% of physiological lymphocytes)		CD45dim, CD19, CD10, CD38, HLA-DR, CD34, Tdt	ALL common
M	CD45, CD2, CD3, CD5, CD7, CD10, CD11a, CD11b, CD11c, CD13, CD14, CD15, CD16, CD19, CD20, CD33, CD34, CD38, CD56, CD58, CD64, CD99, CD117, HLA-DR, TdT, MPO	Major part		CD45dim, CD7, CD34, CD99, CyCD3	ALL T

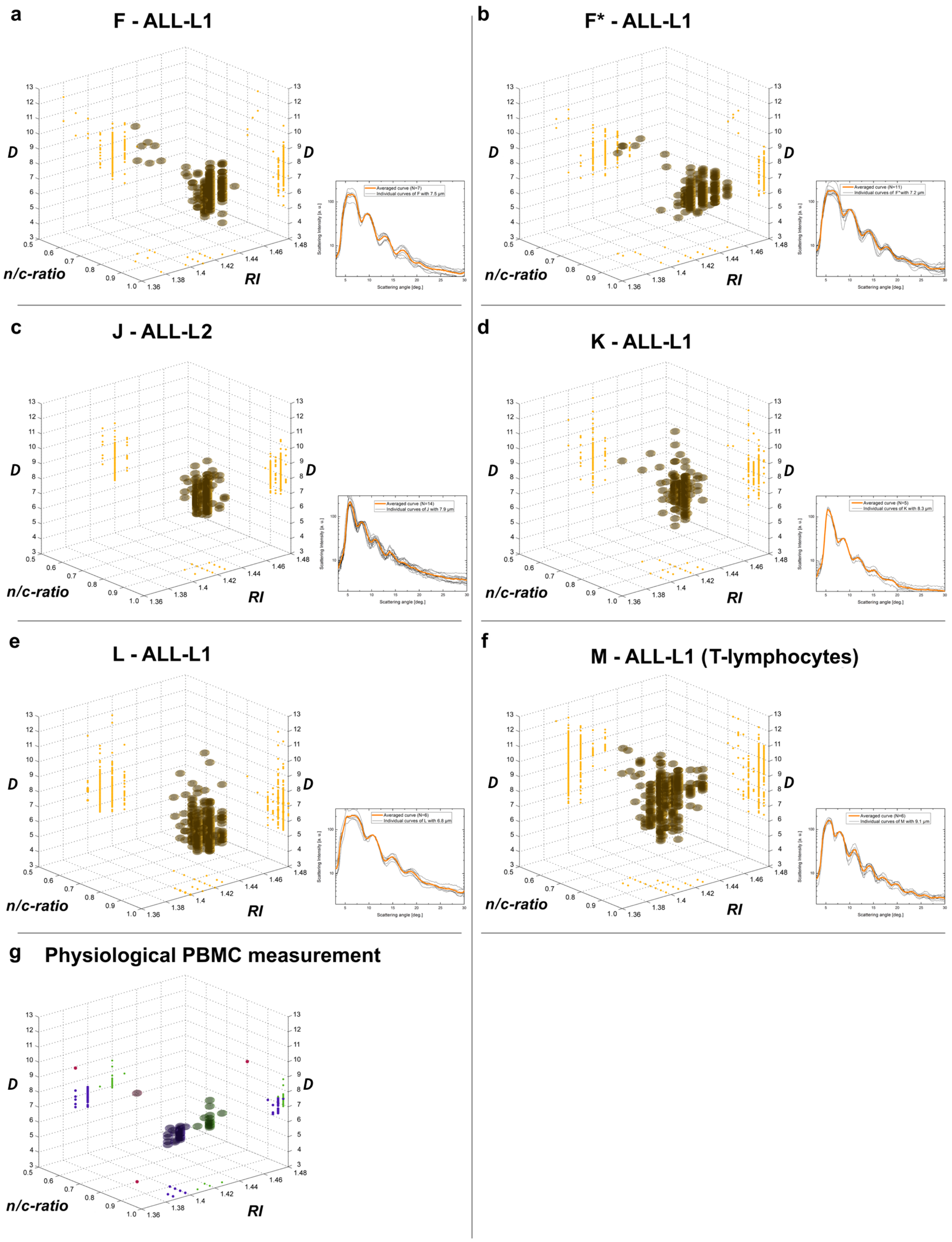

Supplementary Figure 1. Multiple biophysical property results of pathological blood samples and one physiological blood sample are shown. (**a**) An ALL-L1 case of a patient for leukemic B-lymphocytes obtained from peripheral blood is shown, while in (**b**) a blood sample from the bone marrow of the same patient is presented. (**c**) An AML-L2 case (pro-B) is presented, where cells appear slightly smaller compared too other AML-L2 results. (**d**) and (**e**) show other AML-L1 cases. (**f**) An AML-L1 case of leukemic T-lymphocytes is presented, where significant lower RI and higher D values compared to leukemic B-lymphocytes are detected. (**g**) Result of a single PBMC run of 120 seconds is presented (Supplementary Video 2). Each class of PBMC is colored separate for easier readability (blue for T-lymphocytes, green for B-lymphocytes and red for monocytes).

Cluster analysis:

In addition, a cluster analysis for all different types of physiological cells (PBMC classes) as well as all obtained pathological blood samples was performed, using the k-means clustering partitions approach. Hereby k describe the mutually exclusive clusters of the analyzed data. In general, these techniques assign each obtained optical signature result (biophysical cell properties - D, n/c-ratio and RI) to a specific cluster by minimizing the distance from the data point to the mean location of its assigned cluster. For the following examples (Supplementary Figure 2-4), k was chosen according to the expected cell types in the analyzed blood sample. In the case of pathological blood samples, a certain number of physiological data was added to the input data, to better investigate the differences existing between physiological cells and pathological ones. Data from donor D were used for physiological T-, B-lymphocyte and monocyte investigations (463, 123 and 16 cells, respectively), while for pathological cell cluster analysis a certain amount of physiological cells was added to the data, using 463 T-lymphocytes (ID = D), 123 B-lymphocytes (ID = D) or 82 monocytes (ID = B-E). Moreover, the normal distribution of all obtained cell clusters was analyzed, using a paired-sample t-test approach.

Supplementary Table 4. Cluster analysis outcomes for physiological and pathological blood samples, using k-means approach. In the case of pathological blood samples, a certain number of physiological data was added to the input data. Data from donor D was used for physiological T- and B-lymphocyte investigations, while for monocytes data from donor B-E were used (463 T-lymphocytes (ID = D), 123 B-lymphocytes (ID = D) or 82 monocytes (ID = B-E)). The normal distribution of all obtained cell clusters was analyzed, using a paired-sample t-test approach, showing $p<0.01$ and $h=1$. The superimposition of the cluster by cells of other types is expressed in the last column.

ID	*Type*	*Cluster*	*n/c-ratio**	*RI**	*D[µm]**	*Number*	*Superimposed*
D	PBMC - B	D - 1	0.974	1.42	7.34	121	2%
D	PBMC - M	D - 2	0.773	1.39	8.96	16	0%
D	PBMC – T	D - 3	0.950	1.40	6.75	465	1%
F	ALL-L1	F - 1	0.973	1.42	7.81	158	11%
F	ALL-L1	F - 2	0.850	1.39	10.20	6	0%
F	PBMC – B	F - 3	0.972	1.42	7.04	179	41%
F‡	ALL-L1	F* - 1	0.973	1.43	7.20	121	20%†
F‡	ALL-L1	F* - 2	0.825	1.40	10.20	4	0%
F‡	PBMC – B	F* - 3	0.971	1.42	7.24	163	39%†
G	AML-M5	G - 1	0.958	1.41	8.46	592	1%
G	PBMC – M	G - 2	0.796	1.39	9.30	85	6%
H	ALL-L1	H - 1	0.890	1.42	6.98	158	7%
H	ALL-L2	H - 2	0.817	1.40	9.69	46	0%
H	PBMC – B	H - 3	0.972	1.42	7.31	134	8%
I	AML-M1	I - 1	0.836	1.42	8.61	99	43%
I	PBMC – M	I - 2	0.806	1.40	9.09	162	49%
J	ALL-L2	J - 1	0.927	1.42	8.06	147	6%
J	PBMC – B	J - 2	0.971	1.42	7.25	138	17%
K	ALL-L1	K - 1	0.930	1.41	8.49	130	5%
K	PBMC – B	K - 2	0.971	1.42	7.25	135	14%
L	ALL-L1	L - 1	0.965	1.42	6.72	211	13%
L	PBMC – B	L - 2	0.967	1.42	7.39	164	42%
M	ALL-L1	M - 1	0.948	1.41	9.66	206	0%
M	PBMC – T	M - 2	0.948	1.40	6.84	537	14%

* Median values.
† Cluster is normal distributed with a p-value of < 0.1.
‡ Blood sample from the bone marrow.

The following multiple biophysical property graphs visualize typical cluster analysis results of the Supplementary Table 4 for a physiological PBMC case (patient D shown in Supplementary Figure 2), such as for an acute myeloid leukemia (patient G shown in Supplementary Figure 3) and an acute lymphoid leukemia (patient H shown in Supplementary Figure 4) case. Each detected cell cluster was plotted separately according to its different biophysical properties, where graphs from patient G, clearly reflects the presented results of Supplementary Table 4. In fact, physiologic and pathologic monocytes has been differentiated according to their different biophysical properties (also validated with t-student statistical approach), shown in Supplementary Fig. 3. In the case of patient H, physiologic and pathologic lymphocytes have been clearly differentiated. Moreover, the different pathological cell stages were detected in Supplementary Fig. 4 by the cluster analysis routine.

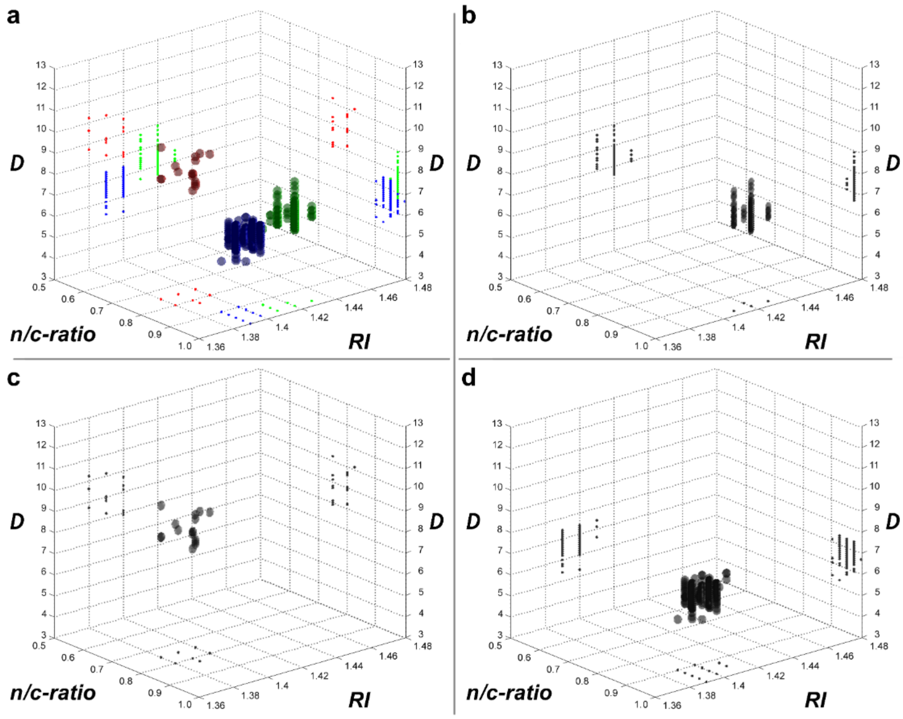

Supplementary Figure 2. Cluster analysis of multiple biophysical property results from physiological PBMC (ID = D). (**a**) Biophysical properties outcomes, before the cluster analysis. Each PBMC class is colored differently for easier readability (blue for T-lymphocytes, green for B-lymphocytes and red for monocytes). (**b**) Detected cluster D - 1 obtained out of all PBMC, shows 98% agreement with the originally detected B-lymphocytes. (**c**) Detected cluster D - 2 shows 100% agreement with the originally detected monocytes. (**d**) Detected cluster D - 3 shows 99% agreement with the originally detected T-lymphocytes. In conclusion, 4 T-lymphocytes out of 602 cells were differently detected as B-lymphocytes by the cluster analysis routine, showing a good agreement.

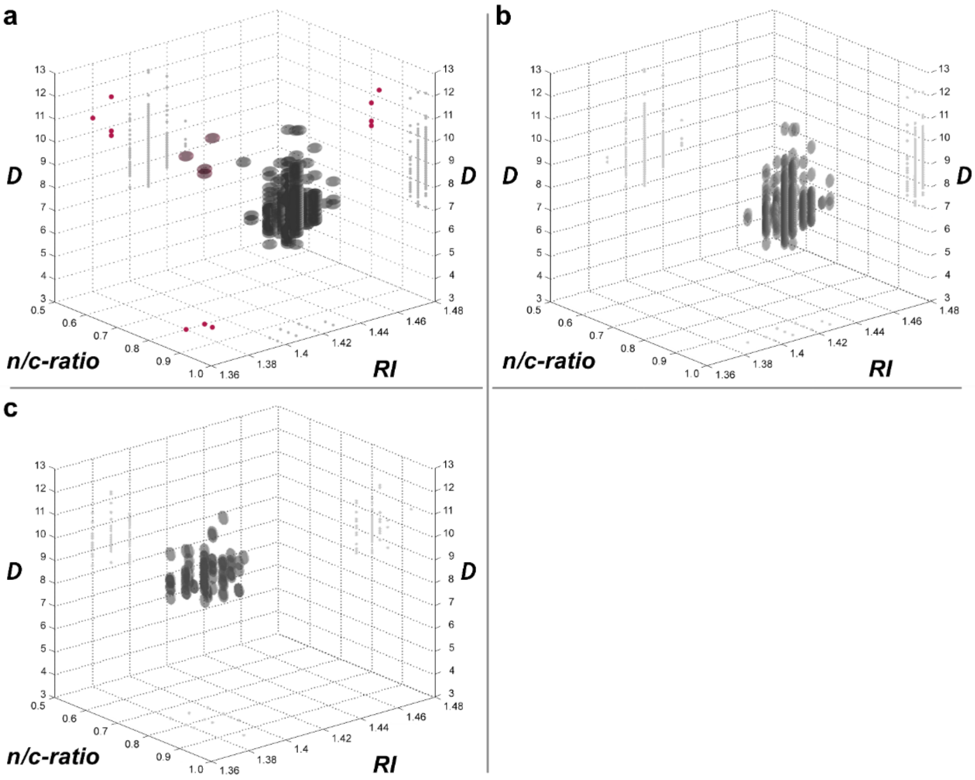

Supplementary Figure 3. Cluster analysis of multiple biophysical properties results from a pathological blood sample (ID = G) mixed with physiological monocytes (ID = B-E) was performed. (**a**) Biophysical properties outcomes, before the cluster analysis (ID = G, see Fig. 5b). The monocyte class is colored in red. (**b**) Detected cluster G - 1 obtained out of all cells, shows 99% agreement with the originally detected pathological B-lymphocytes. (**c**) Detected cluster G - 2 shows 94% agreement with the originally detected monocytes. In conclusion, 3 monocytes out of 677 cells were differently detected by the cluster analysis routine, showing a good agreement.

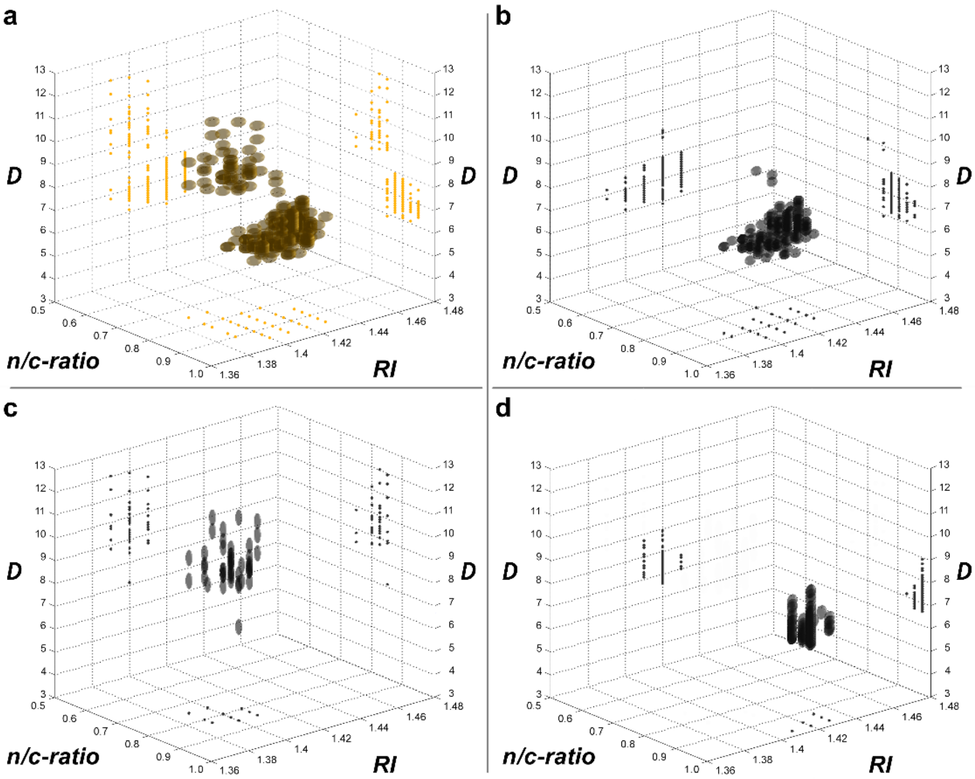

Supplementary Figure 4. Cluster analysis of multiple biophysical properties results from a pathological blood sample (ID = H) mixed with physiological B-lymphocytes (ID = D) was performed. (**a**) Biophysical properties outcomes, before the cluster analysis (ID = H, see Fig. 5d). (**b**) Detected cluster H - 1 obtained out of all cells, shows 93% agreement with the originally detected pathological B-lymphocytes case L1. (**c**) Detected cluster H - 2 shows 100% agreement with the originally detected pathological B-lymphocytes case L2. (**d**) Detected cluster H - 3 shows 92% agreement with the added physiological B-lymphocytes. In conclusion, 11 pathological B-lymphocytes out of 338 cells were differently detected by the cluster analysis routine, showing a good agreement.
